# Supplementary material for: Complex bacterial diversity of Guaymas Basin hydrothermal sediments revealed by synthetic long-read sequencing (LoopSeq)
Source: Front Microbiol. 2025 Jan 7;15:1491488. doi: 10.3389/fmicb.2024.1491488 (PMC11747844; doi:10.3389/fmicb.2024.1491488)
Supplement: Supplementary file 1 [file Data_Sheet_1.docx]

**DNA Extraction protocol**

DNA extraction from Guaymas Basin sediment samples was adapted from a previously published extraction method that combines physical, chemical, and enzymatic steps and was optimized for sediment samples (Zhou, Bruns, & Tiedje 1996). We scaled it down from 3 g sediment to 0.5 g sediment and adjusted all components accordingly.

1.35 ml of DNA extraction buffer (see below) was added to 0.5 g frozen sediment and stirred with a sterile spatula.

**DNA extraction buffer for 300 ml total**

100 mM Tris-HCl, pH 8.0 (adjusted to pH 8.0 with 1 M HCl) 30 ml of 1M stock

100 mM EDTA, pH 8.0 60 ml of 0.5M stock

100 mM sodium phosphate buffer, pH 8.0 60 ml of 0.5M Stock

1.5 M NaCl 90 ml of 5M Stock

1% CTAB 3 g to 300 ml

Physical extraction:

The slurry was frozen in at -20°C and thawed thoroughly in a water bath at 65°C. Freezing and thawing was repeated three times.

Biological extraction:

After the slurry cooled down to 35°C, 5 μl of Proteinase K (20 mg ml-1) was added and samples incubated for 30 min at 37°C under mild horizontal shaking. This step can be extended ad lib, e.g., to an overnight incubation with enzyme. (For difficult samples, e.g., containing many gram-positive bacteria it is advised to include an incubation with Lysozyme before the Proteinase step; we note that this additional step was not used with our Guaymas Basin samples).

Chemical extraction:

0.3 ml of 10% sodium-dodecylsulfate (SDS) was added and incubated (2 hours, 65°C) with gentle end over end inversion every 25 min. The slurry was centrifuged (3220 *g* for 10 min at RT) and the supernatant collected.

To increase yield, an additional extraction step can be used, but was not performed here. In this step (described for 3 g sediment), the pellet was again treated with 2.7 ml DNA extraction buffer and 0.6 ml 10% SDS, incubated for 10 min at RT and centrifuged (3220 *g* for 10 min at RT). Supernatants were combined (~11 ml), and samples can be split as needed to the final organic extraction; an equal volume of chloroform/isoamylalcohol (24:1) was added and the mixture was shaken gently, but thoroughly. After centrifugation (3220 *g* for 10 min at RT) the aqueous phase was collected and 0.6 volumes of isopropanol (abs.) were added to precipitate the DNA (overnight, 4°C).

Pellet washing

The pellet was washed with cold ethanol (80% v/v), centrifuged (20,000 *g* for 10 min at 4°C), dried (15 -30 min at RT) and gently resuspended (1 hour at 4°C, no pipetting) in a suitable volume (50 µl – 250 µl) of 0.5× TE or PCR-water. If CTAB crystals form in the precipitate, the suspension was warmed up in a water bath (1 min at 70°C) to dissolve precipitated CTAB, centrifuged (20,000 *g* for 25 min at RT) and the supernatant discarded.

Pellet clean-up:

The DNA suspension was purified using the Wizard DNA clean-up system, which is based on the adsorption and desorption of DNA to a matrix. The DNA was eluted twice with 25 μl 0.5× TE (65 °C) and the yield was checked by gel electrophoresis with Sybr Red gel stain. Gel electrophoresis was carried out with agarose gels (1% agarose in 1× TAE) in Bio-Rad electrophoresis chambers containing 1x TAE at an electrical current of 8-10 V cm-1. Pockets were loaded with 2 μl of DNA extract (or low DNA mass ladder) mixed with 1 μl of 6× loading buffer. Fluorometric measurements of DNA concentrations were performed with Thermofisher Scientific Qubit and Nanodrop instruments.

**Reference**: Zhou J, Bruns MA, Tiedje JM. 1996. DNA recovery from soils of diverse composition. Applied and Environmental Microbiology. 62:316-22.

**Images displaying the color gradient of chromophoric biomass in Guaymas Basin sediments after organic extraction and centrifugation, but before the removal of the organic phase. Note the removal of chromophoric biomass with depth and hydrothermal influence.**

*
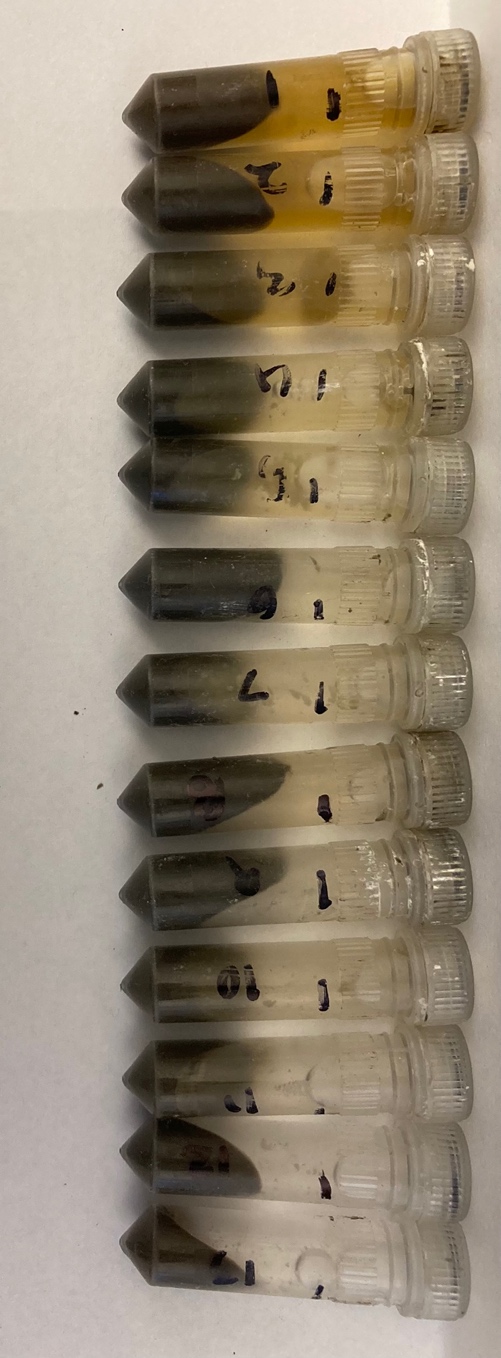
*

Bare sediment site samples (4872-01). Samples arranged by depth left to right (leftmost sample = top layer of sediments, rightmost sample = bottom layer of sediments).

*
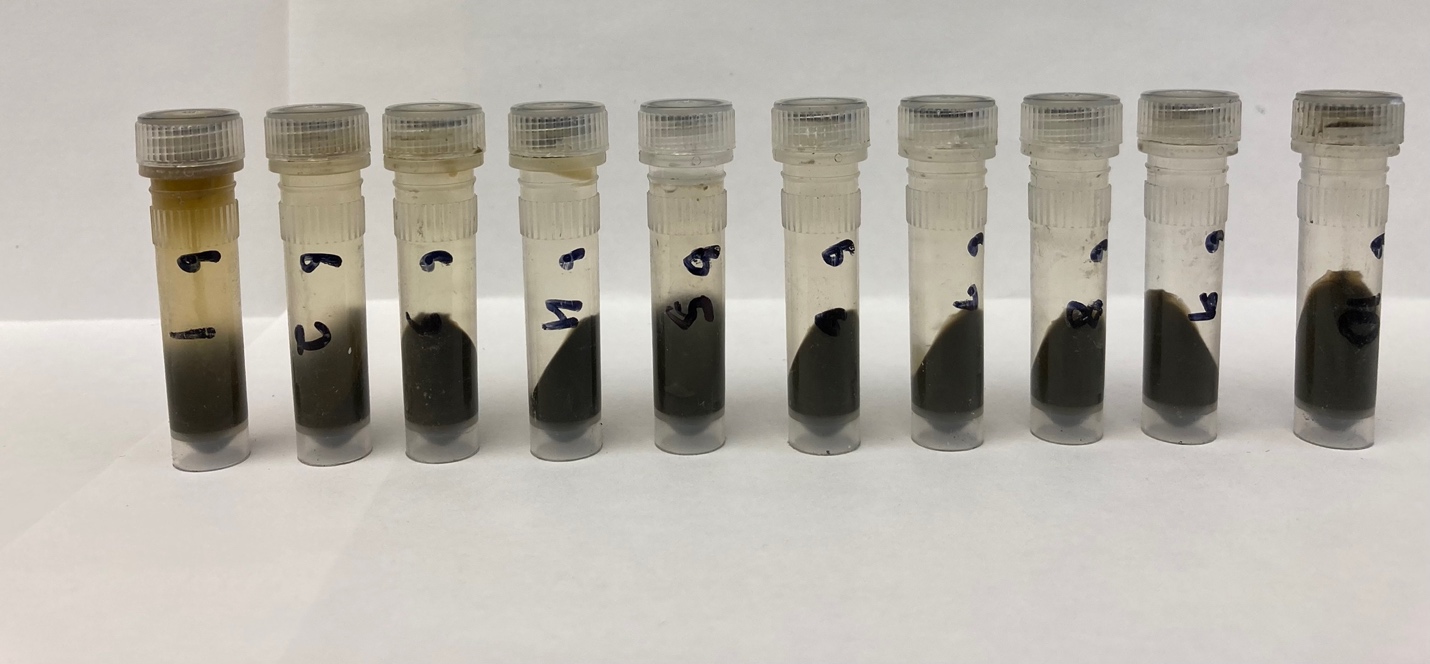
*

White mat sediment site samples (4872-06). Samples arranged by depth left to right (leftmost sample = top layer of sediments, rightmost sample = bottom layer of sediments).

*
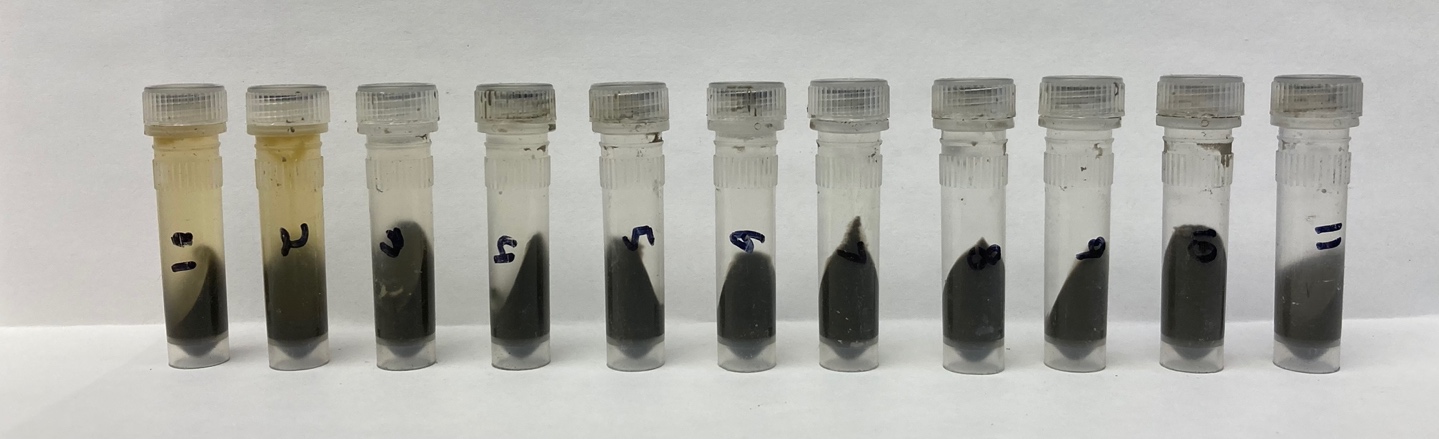
*

Orange mat sediment site samples (4872-14). Samples arranged by depth left to right (leftmost sample = top layer of sediments, rightmost sample = bottom layer of sediments).
