# Supplementary material for: Complex bacterial diversity of Guaymas Basin hydrothermal sediments revealed by synthetic long-read sequencing (LoopSeq)
Source: Front Microbiol. 2025 Jan 7;15:1491488. doi: 10.3389/fmicb.2024.1491488 (PMC11747844; doi:10.3389/fmicb.2024.1491488)
Supplement: Supplementary file 4 [file Data_Sheet_4.docx]

**Supplementary Figures**

Complex bacterial diversity of Guaymas Basin hydrothermal sediments revealed by synthetic long-read sequencing (LoopSeq)

John E. Hinkle, Jeffrey P. Chanton, Molly A. Moynihan, S. Emil Ruff, Andreas Teske

**Supplementary Figure 1.** Histogram of sequence read lengths (in base pairs) as generated by the 27F and 1492R 16S rRNA gene sequence primers utilized by LoopSeq.

**Supplementary Figure 2.** Alpha diversity figure using the Observed, Chao1, Shannon, Simpson, and Inverse Simpson metrics for the sequencing datasets obtained at the three coring sites (Core 4872-01: the bare sediment site, Core 4872-06: the white mat site, and Core 4872-14: the orange mat site). No significant difference in alpha diversity between sites was observed for any metric (Observed: p = 0.7656, Chao1: p = 0.3432, Shannon: p = 0.7537, Simpson: p = 0.2759, Inverse Simpson: p = 0.2759). Samples with < 400 reads were not included in the analysis. Normalization was performed by subsampling to 400 reads.

**Supplementary Figure 3.** Dendrogram plot using the Bray-Curtis dissimilarity method. Samples with < 400 reads were not included in the analysis.


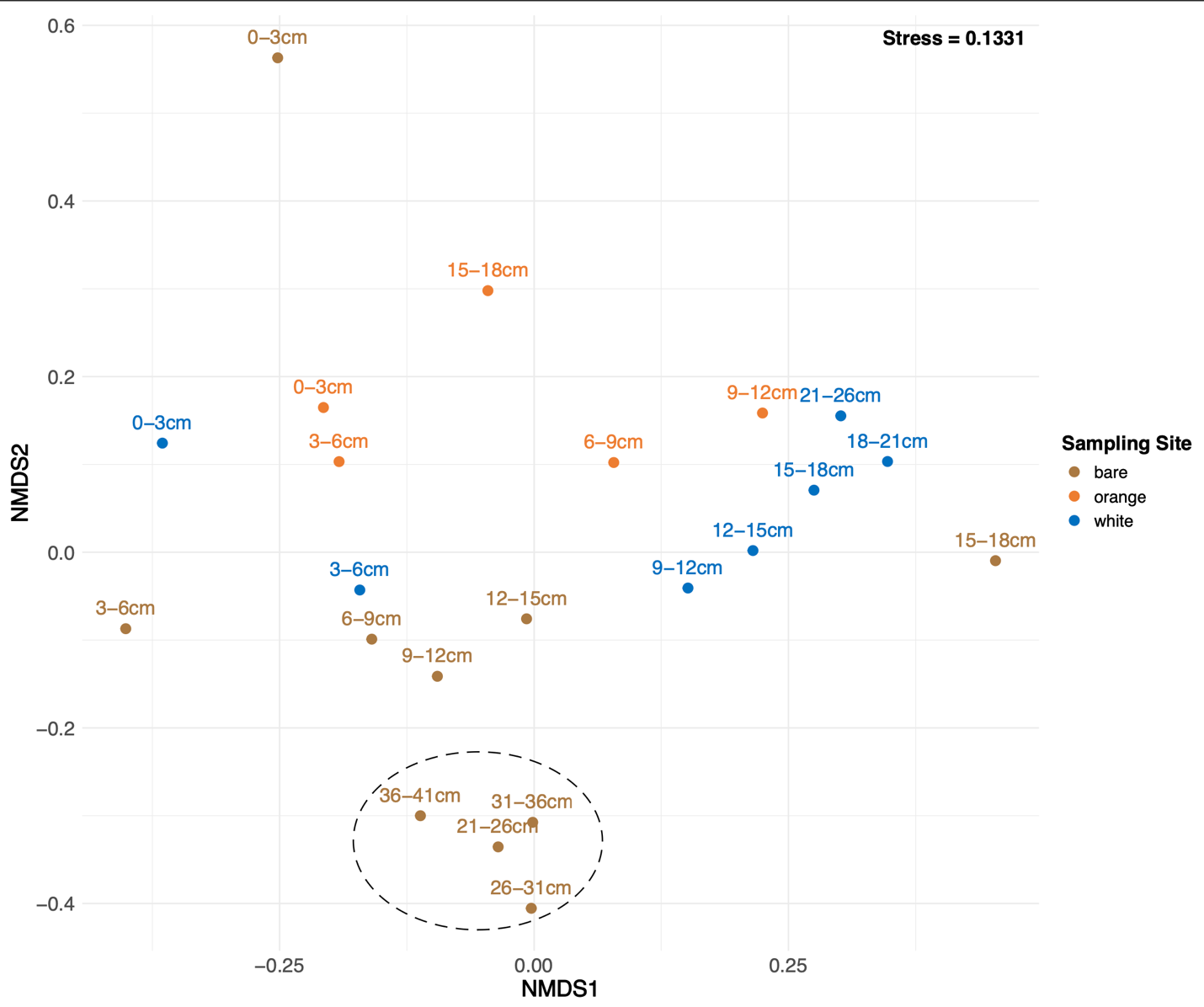


**Supplementary Figure 4.** Non-metric multi-dimensional scaling (NMDS) plot using the Bray-Curtis dissimilarity method. “Deep” (≥ 21cmbsf) bare sediment site cluster highlighted with dashed line. Samples with < 400 reads were not included in the analysis.


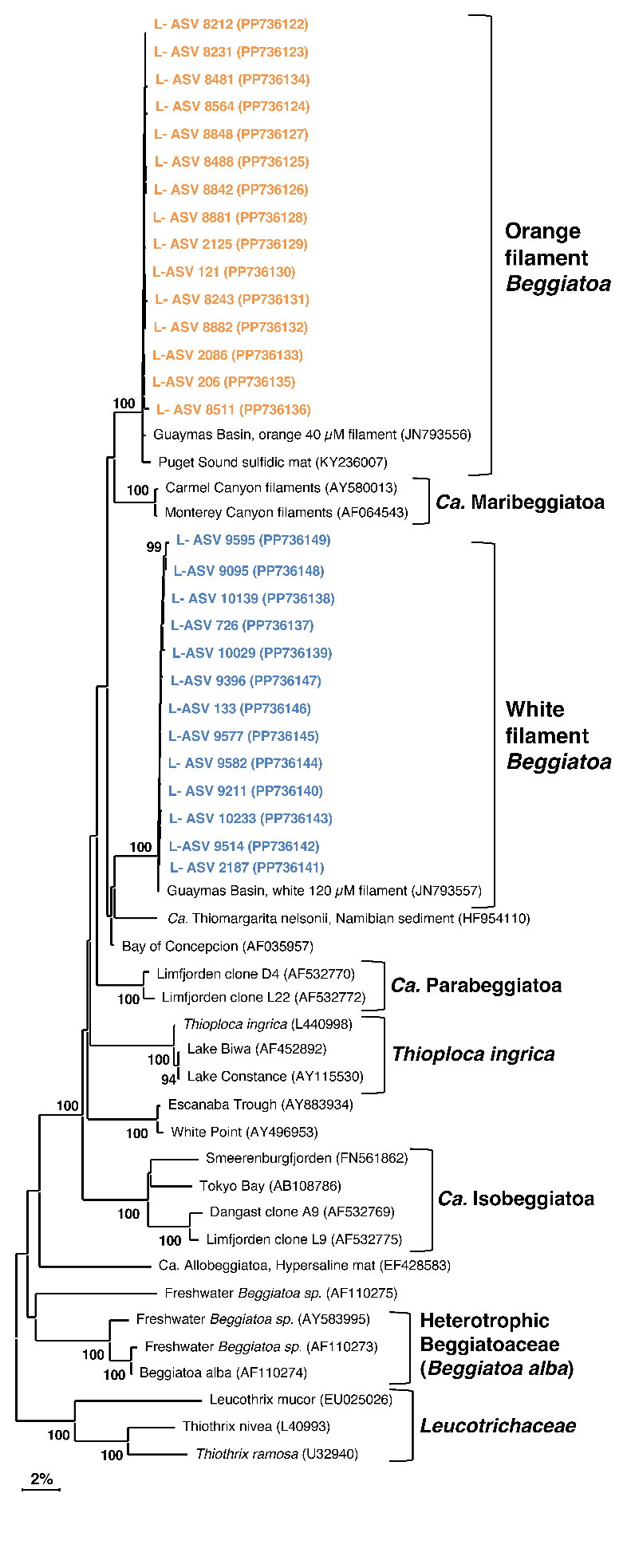


**Supplementary Figure 5.** Expanded distance phylogeny of *Beggiatoaceae* (rooted by *Leucotrichaecea*) depicting all L-ASVs recovered at each mat site (orange, white).


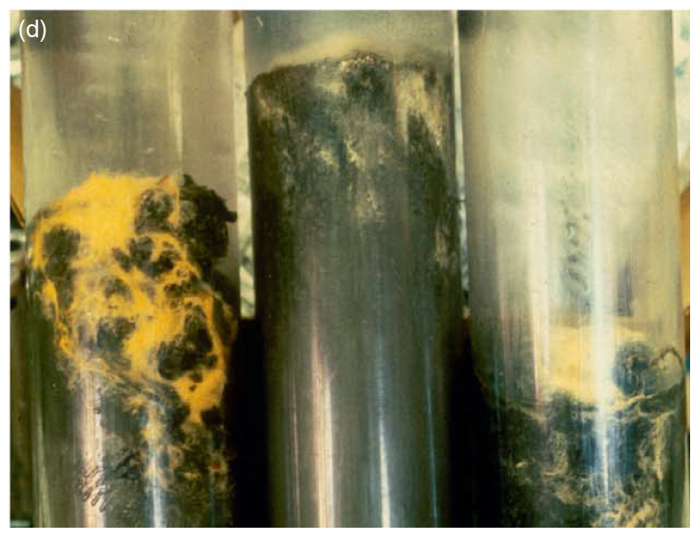


**Supplementary Figure 6.** Image of sediment push cores from Guaymas Basin. The image depicts how *Beggiatoaceae* filaments can be pulled deeper into the sediment column during coring and lead to the recovery of *Beggiatoaceae* in subsurface sediments. The image is from Teske, A., 2009. Deep sea hydrothermal vents. P. 346-356. In: *Desk Encyclopedia of Microbiology (Ed. M. Schaechter) Academic Press*.).

**Supplementary Figure 7.** Distance phylogeny of *Thermodesulfobacterium* and related lineages.

*Thermodesulfobacterium torris 16S rRNA sequence was obtained from annotated genome (BioSample ID: SAMN27514933).

**Thermodesulfobacterium syntrophicum 16S rRNA sequence was obtained from annotated genome (BioSample ID: SAMN29995626).
